# Virtual Engagement in a Social Media Community of Mothers With Substance Use Disorders: Content Analysis

**DOI:** 10.2196/24353

**Published:** 2021-06-24

**Authors:** Shayna Mazel, Yaara Zisman-Ilani, Shannon Hennig, Deborah Garnick, Joanne Nicholson

**Affiliations:** 1 Heller School for Social Policy and Management Brandeis University Waltham, MA United States; 2 College of Public Health Temple University Philadelphia, PA United States; 3 Maternal Mental Health Research Collaborative Calgary, AB Canada

**Keywords:** virtual engagement, virtual community participation, social media, mental health, opioids, substance use

## Abstract

**Background:**

Co-occurring substance use disorder is common among pregnant and parenting women with mental illness, but their engagement with and utilization of relevant services and treatment is low. Social media has the potential to convey benefits and facilitate engagement among this target group.

**Objective:**

This study aimed to explore the reach and engagement of specific social media posts among pregnant women and mothers with substance use disorders.

**Methods:**

Eighteen posts providing content related to substance use (cannabis, opioids, or alcohol), varying in type of content (informational or experiential) and target (policy-, practice-, or perception-related), were posted in a closed Facebook community page comprising over 33,000 pregnant women and mothers between May 2019 and October 2019.

**Results:**

The overall level of reach of these Facebook posts ranged from 453 to 3045 community members. Engagement levels, measured via the number of likes, comments, or posts shared, varied based on the type of post content (ie, informational or experiential).

**Conclusions:**

Participation in a virtual community via social media platforms can facilitate engagement among pregnant women and mothers with mental illness by communicating relevant information about substance use, as well as potentially promoting awareness of, access to, and engagement with treatment services.

## Introduction

During the perinatal period, women are vulnerable to a variety of life stressors and are at a higher risk of experiencing depressive symptoms [1,2]. Many find themselves at an increased risk for using substances [3-5]. Yet, women’s needs for mental health services are often unmet [6], and those who are vulnerable and at high risk often disengage from mental health services and substance use treatment [7,8]. Several factors contribute to such high levels of disengagement from services and treatment among high-risk women, including stigma, fear of losing the child’s custody, or fear of legal consequences of substance use [3,9-14]. The postpartum period itself presents additional stressors such as childcare responsibilities, work-life balance, and physical and emotional recovery, which may interfere with the utilization of mental health and/or substance use services [15,16].

Social media communities have become a central platform for individuals seeking emotional support, especially during the COVID-19 pandemic [17,18]. Social media communities are web-based platforms wherein members with common experiences and interests share real‐time information and offer support [19-22]. Social media offers anonymity, reduces perceptions of stigma, and helps eliminate geographic barriers [20,23]. Therefore, by providing relevant information and support, social media has the potential to overcome barriers to facilitate engagement among women during pregnancy and in the postpartum period [24,25].

Recent surveys indicate that individuals experiencing bipolar disorder, major depressive disorder, anxiety, or schizophrenia spectrum disorder are inclined to using Facebook [26]. However, little is known about the willingness of social media users and online community members to engage interactively in substance use–related topics on social media platforms. Furthermore, limited information is available about the potential of those online communities for engaging pregnant and parenting women with mental illness who are at risk for using substances. The purpose of this exploratory study is to fill a gap in the literature by investigating, for the first time, the extent to which social media posts can be used to increase outreach to high-risk, vulnerable women. We also investigated how social media posts can increase awareness and sense of support among women and facilitate engagement in managing their mental health. Specifically, this study focuses on the level of *reach* of social media posts, impact of the post content, and relevance of the target of content to the *engagement* within an online community.

## Methods

### Study Design

**Figure 1:**
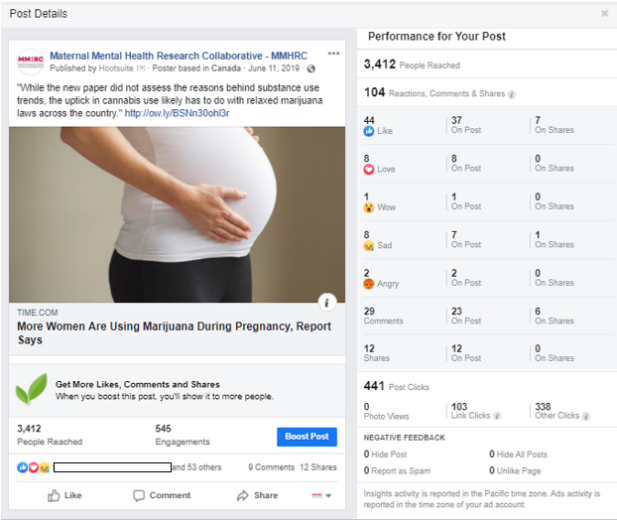
An example of a cannabis-related Facebook post on the Maternal Mental Health Research Collaborative Facebook page.

**Figure 2:**
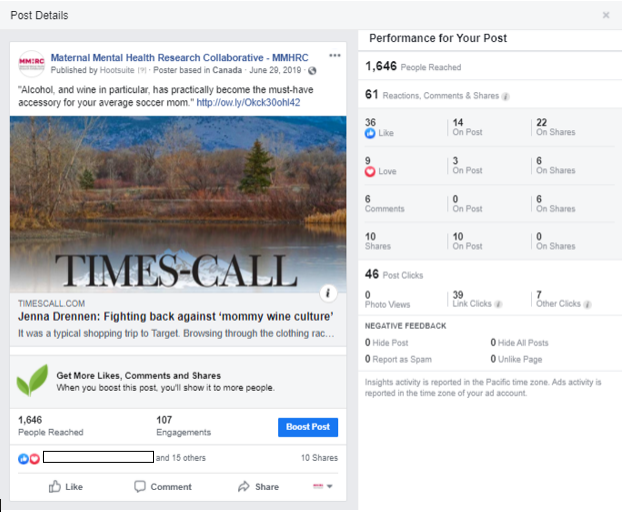
An example of an alcohol-related Facebook post on the Maternal Mental Health Research Collaborative Facebook page.

This exploratory, descriptive study assessed responses to Facebook posts pertaining to substance use (N=18) on the Maternal Mental Health Research Collaborative (MMHRC) Facebook page [27] between May and October 2019. Data were obtained from the MMHRC Facebook page by using Facebook Insights data in response to the purposefully selected sample of posts regarding substance, type, and target of content. Examples of published posts can be seen in Figures 1 and 2. This study was deemed exempt by the Brandies University Institutional Review Board.

### Setting

MMHRC is a peer-led, patient-centered online community dedicated to linking pregnant and parenting women with mental health conditions with information and support, providers, and researchers [28]. Currently funded by the Patient Centered Outcomes Research Institute (PCORI), the MMHRC was created in 2016 to provide opportunities for mothers, researchers, providers, and other stakeholders to cocreate research; amplify the voices of women who have experienced a perinatal mood or anxiety disorder; and foster strength, advice, and support within a community of others with shared experiences. MMHRC is an active group in many social media networks, including Facebook, Twitter, Instagram, and Pinterest, and it utilizes social media best practices to expand its reach.

The MMHRC Facebook page has an international base of followers or Facebook users who choose to receive updates from the MMHRC Facebook page. Ninety percent of MMHRC’s followers are from the United States and Canada, with additional followers living in Australia, the United Kingdom, South Africa, India, Philippines, New Zealand, Ireland, and Mexico. During the time of the study (May 2019 to October 2019), the MMHRC Facebook page following rose to 32,410 followers, and it currently exceeds 33,000 followers. The vast majority are women (99%). Of those identifying as women, 92% were of childbearing age (18-44 years old) at the time they joined the community. Followers of the MMHRC Facebook page do not need to self-identify as having a mental illness in order to view page content. We posit that these followers are users who are interested in or may have a lived experience of mental illness. Because extensive research in the literature underscores the high prevalence of co-occurring mental health and substance use disorders, we conjecture that many women with mental illness may have experience with substance use issues as well [29-32].

### Study Sample: Facebook Posts

In this study, the analytic sample comprised 18 Facebook posts selected by the researchers to allow exploration of the relationship between the post’s characteristics and potential reach and engagement. A Facebook post was defined as content that is inserted and appears on a Facebook newsfeed. For the MMHRC Facebook page, posting is managed by the page administrator—the MMHRC Program Director. Most posts are accompanied by a caption, also inserted by the page administrator, as an introduction to or an explanation of the content posted (eg, a web link to an article, photo, or graphic). Content on the MMHRC Facebook page is posted approximately four times a day—at 7:00 am, 9:00 am, 12:00 pm, and 2:00 pm Mountain Time (MT). The study content was posted on the MMHRC Facebook page on randomly selected days and times during the study months. The purposefully selected sample of substance use–related posts were considered independent variables in this study. These posts included content selected by members of the research team from the scientific literature, gray literature, and popular press, during the study months. To help generate interest in the content among Facebook users, all content was purposely designed to be relatively current, with most posts from content that was recently published. Each post was categorized in three ways: (1) by specific substance (opioids, cannabis, or alcohol), (2) the type of information delivered (informational or experiential), and (3) the target of the post (policy-, practice-, or perception-related). Informational posts included content that educates and informs. Examples of informational posts included a research article providing statistics about opioid use among pregnant women or information pertaining to a recently opened recreational cannabis facility. Experiential posts included content that was designed to conjure sensory and behavioral reactions or material that may be relatable to followers’ experiences with substances [33]. Examples of experiential posts included a woman’s blog post about her experience in recovery from using opioids or a link to popular media article about a celebrity’s story of alcohol use while they were pregnant. Policy-related posts reflected aspects of local, state, or federal policy pertaining to substance use. Practice-related posts referred to an individual’s actual use of opioids, cannabis, or alcohol (ie, a person’s behavior or habits). Perception-related posts referred to individual, organizational, or societal beliefs, awareness, or judgement about opioid, cannabis, or alcohol use, treatment, and recovery. Members of the research team identified and discussed each post and achieved consensus regarding the categorization of post characteristics. It is important to note that the titles of posts do not fully reflect their content; content was reviewed as part of the categorization process (Table 1).

**Table 1:** Characteristics of Facebook posts classified by substance.

Substance type and post title		Date posted (2019)	Type	Target
**Opioids**				
	Insurance and pregnancy are barriers to opioid treatment	June 1	Informational	Policy
	Opioids increasingly tied to deaths of pregnant women	May 14	Informational	Practice
	Embracing the needs of pregnant women and infants in our nation's battle against the opioid crisis	May 24	Informational	Perceptions
	Most physician health plans don't allow medical professionals access to the same treatment as patients	September 30	Experiential	Policy
	The dangerous stigma against pregnant women addicted to opioids	July 10	Experiential	Practice
	How the opioid epidemic affects women differently	June 13	Experiential	Perceptions
**Cannabis**				
	Is cannabis safe during pregnancy? More expecting mothers are wondering	August 7	Informational	Policy
	Pregnant women in the US are giving up major vices—except one	May 15	Informational	Practice
	Marijuana use doubles in U.S. pregnant women, especially during first trimester	August 9	Informational	Perceptions
	More women are using marijuana during pregnancy, report says	June 11	Experiential	Policy
	Why some mothers keep using cannabis during pregnancy and breastfeeding	June 19	Experiential	Practice
	Good Chemistry, Worcester’s first marijuana dispensary, is open for recreational sales	May 5	Experiential	Perceptions
**Alcohol**				
	Conversational approach most successful way of encouraging drinking habit disclosure	October 1	Informational	Policy
	Study tracks drinking habits of new parents	June 4	Informational	Practice
	Mixed messages about safe consumption during pregnancy	September 30	Informational	Perceptions
	Drinking during pregnancy: is a little alcohol ever okay?	June 6	Experiential	Policy
	Jenna Drennen: fighting back against ‘mommy wine culture’	June 29	Experiential	Practice
	Does pregnant Gretchen Rossi drink a little wine?	June 7	Experiential	Perceptions

### Measures

#### Organic Reach

Organic reach was defined as the number of unique Facebook users who viewed an MMHRC post as indicated by Facebook Analytics. Users can view a post that is pushed to their newsfeed or find Facebook content via word of mouth, online or offline referrals, and from targeted web searches of the Facebook page. Organic reach metrics were used in this study rather than total reach metrics that include paid reach, to reflect the number of unique users reached by each piece of content. Although organic and paid reach data together indicate the number of Facebook users who saw or engaged with a post and both metrics are a result of Facebook-created search algorithms, organic reach considered is a better indicator of the number of users who may have specifically sought out MMHRC-related content. In contrast, paid reach includes the number of Facebook users who may have seen MMHRC content because it was provided to them by paid Facebook advertising. Facebook does not publish information regarding how they operationalize their algorithms.

#### Engagement

Engagement was measured in terms of actions taken by the Facebook user in response to viewing content—an original post, a shared post, or another user’s comment regarding a post. Engagement data, also obtained via Facebook Analytics, included the following:

- The number of *clicks* was defined as the total number of times people click on a post content.
- The number of *likes* was defined as the total number of times people like either an original post content or a comment by another user.
- The number of *comments* was defined as the total number of times users respond to posts by entering text into a post’s comment box, including emojis, tags, or hashtags (eg, @JohnDoe or #PMAD).
- The number of *shares* was defined as the total number of times users share content on their personal timeline; a Facebook friend’s timeline; in a Facebook group; in an event page; in a page managed by the user; or in a private message to a friend, group, or page.

Facebook provides a dashboard of quantitative information regarding user views or the number of users reached by each Facebook post, as well as the number of times content was reshared by users. User views are only viewable by the administrators of the Facebook account, whereas the volume of engagement for each post (ie, clicks, likes, comments, and shares) are public.

Facebook discloses the number of *likes* garnered from each post, as well as emoji-based *reactions*. These reactions include the number of positive reactions as well as negative reactions to the post (eg, sad or angry emojis) to the posts. These data provide insight into the level of engagement elicited by each Facebook post.

### Procedures and Analyses

Members of the research team extracted data using Facebook Analytics, compiling responses to post information into a standard spreadsheet format. Responses to Facebook content were deidentified as data were extracted, to ensure anonymity of Facebook users who view the MMHRC Facebook page was maintained. Data were extracted at least one month after substance use content was posted, as Facebook uses that timeframe when sharing information about reach. Descriptive counts were compiled and tabulated according to substance (cannabis, opioids, or alcohol), content type (informational or experiential), and target (policy-, practice-, or perception-related) to facilitate review by the research team over the course of the study and to report findings.

## Results

### Reach and Engagement Classified by Substance Featured in Post Content

Posts related to cannabis generated the largest reach on Facebook, followed by alcohol- and opioid-related content (Table 2). User reactions to content had a similar ranking; cannabis-related content generated the most reactions, followed by opioid- and alcohol-related content. The level of engagement paralleled the level of reach. Cannabis-related content generated the highest levels of engagement, followed by alcohol- and opioid-related content.

**Table 2:** Levels of reach and engagement per Facebook post classified by substance.

Substance type and post title		Organic reach, n	Clicks, n		Likes, n		Comments, n		Shares, n		
**Opioids**											
	Insurance and pregnancy are barriers to opioid treatment	530	3	4		0		2			
	Opioids increasingly tied to deaths of pregnant women	688	17	8		0		3			
	Embracing the needs of pregnant women and infants in our nation's battle against the opioid crisis	491	10	3		0		1			
	Most physician health plans don't allow medical professionals access to the same treatment as patients	461	4	0		0		0			
	The dangerous stigma against pregnant women addicted to opioids	1117	24	8		1		2			
	How the opioid epidemic affects women differently	833	18	3		2		3			
**Cannabis**											
	Is cannabis safe during pregnancy? More expecting mothers are wondering	1242	125	12		3		3			
	Pregnant women in the US are giving up major vices—except one	1076	56	5		1		1			
	Marijuana use doubles in U.S. pregnant women, especially during first trimester	1990	59	17		0		10			
	More women are using marijuana during pregnancy, report says	3045	441	44		29		12			
	Why some mothers keep using cannabis during pregnancy and breastfeeding	1256	67		9		1		5		
	Good Chemistry, Worcester’s first marijuana dispensary, is open for recreational sales	654	8		5		0		2		
**Alcohol**											
	Conversational approach most successful way of encouraging drinking habit disclosure	453	13		0		0		0		
	Study tracks drinking habits of new parents	2291	249		17		11		12		
	Mixed messages about safe consumption during pregnancy	1086	204		9		0		3		
	Drinking during pregnancy: is a little alcohol ever okay?	754	30		6		0		1		
	Jenna Drennen: fighting back against ‘mommy wine culture’	1642	46		36		6		10		
	Does pregnant Gretchen Rossi drink a little wine?	534	22		2		0		0		

### Reach and Engagement Classified by Type of Post Content

Experiential posts reached more Facebook users than informational posts for cannabis- and opioid-related content, whereas informational posts reached more Facebook users than experiential posts for alcohol-related content (see Tables 1 and 2). A similar trend was observed for the number of likes, comments, and shares; there were more likes, comments, and shares for experiential posts than informational posts for cannabis- and opioid-related content, whereas there were more likes, comments, and shares for informational posts than experiential posts for alcohol-related content.

For cannabis-related content, experiential posts reached and engaged more Facebook users than informational content. For opioid-related content, experiential posts reached more Facebook users and had slightly higher levels of engagement than informational posts. For alcohol-related content, informational posts both reached and engaged more Facebook users than did experiential posts.

### Reach and Engagement Classified by Target of Post Content

For cannabis-related content, policy-targeted posts reached and engaged almost double the number of Facebook users than perception- and practice-targeted posts. Although the levels of reach were similar for practice- and perception-targeted posts, practice-targeted posts had almost double the number of clicks yet half the number of shares and similar numbers of likes as compared to perception-targeted posts. For opioid-related content, practice-targeted posts reached the most Facebook users (n=1805), followed by perception-targeted posts (n=1324) and policy-targeted posts (n=991). Practice-targeted posts had almost double the number of clicks as perception-targeted posts and more than five times the number of clicks as policy-targeted posts. Practice-targeted posts also had more likes, comments, and shares than perception- and policy-targeted posts. For alcohol-related content, practice-targeted posts reached the most Facebook users (n=3933), followed by policy-targeted posts (n=1840) and perception-targeted posts (n=987). Although policy-targeted posts had less than half the level of reach compared to practice-targeted posts, both post types had similar numbers of clicks (n=234 and n=295, respectively). Perception-targeted posts had a much lower number of clicks (n=35). Practice-targeted posts also had more likes and shares than policy- and perceptions-targeted posts.

## Discussion

### Principal Findings

The purpose of this study was to explore, for the first time, the level of engagement with social media posts in an online community for mothers with mental health and substance use disorders. The study supports the potential benefit of social media in reaching mothers and conveying information about substance use, potentially serving as an avenue to treatment access and engagement. Our findings highlight the potential benefits of using social media to provide preventive messaging about substance use during or after pregnancy and opportunities to provide information to women about what is safe and unsafe and how to anonymously ask these questions.

Social media posts through the MMHRC Facebook page reached approximately 3000 members of the online community. Although previous studies have not explored whether different substances or content characteristics elicit different levels of reach and engagement, our results suggest that social media users who viewed the posts may have been more interested in engaging with content related to cannabis use than alcohol or opioid use at the time of the study. It is possible that cannabis elicited more reach and engagement among these women because cannabis use has increased among women and pregnant women in recent years [34] and because, at the time of the study, additional states decriminalized cannabis, possibly resulting in additional media coverage and awareness. With a focus on cannabis-related content, policy-targeted posts garnered more reach and engagement than practice- and perception-targeted posts. For opioid- and alcohol-related content, however, posts that were targeted toward practice generally garnered more reach, clicks, likes, comments, and shares than those targeted toward policy or perceptions. Cannabis- and opioid-related experiential posts also generated more reach and engagement than informational posts, as compared to alcohol-related content (which generated more reach and engagement from informational posts). These results have implications for which types of content may be best shared in a social media setting. Perhaps it would behoove individuals looking to share substance use content to tailor posting language in accordance with these findings, acknowledging that women may be more likely to engage with content that pertains to more widely used substances (eg, cannabis). At the same time, these findings suggest that women are interested in information pertaining to alcohol use and perhaps would further engage with informational social media posts.

### Limitations

There are a few notable limitations to this exploratory study. First, this study refers to substance use or misuse as a continuum of substance use problems. We have not made a distinction between substance use or misuse and substance use disorders, such as meeting the Diagnostic and Statistical Manual of Mental Disorders criteria (which is less common than use or misuse), although using or misusing substances during pregnancy requires different strategies (ie, preventative messaging) as opposed to substance use disorders (which implies chaotic or uncontrolled use despite consequences). This study also presents the results of online engagement with one social media–based community; therefore, the findings may not be generalizable to other online communities and to the general population of Facebook users. As the MMHRC’s mission is to connect mothers with researchers and other stakeholders in the cocreation of research, it is possible that MMHRC followers are more willing to be engaged in content than similar online communities and social media–based peer support networks. At the same time, MMHRC followers do not need to self-identify with a mental illness or have experience with substance use or substance use disorder in order to view or engage with the Facebook community posts. Hence, viewing or engaging with the posts does not necessarily suggest that a person is actively engaged in substance use, meets the criteria for a substance use disorder, or is interested in treatment or other health services.

Second, only one piece of content was posted for each intersection of substance type, type of post, and target of post. Since only 18 pieces of content were posted in total, the generalizability of findings is further limited, as one piece of substance use content cannot fully represent all of its intersections. Moreover, although the time of posting was randomized, each piece of content was not posted more than once. Therefore, the day and time of posting could have impacted the levels of reach and engagement for each post.

Third, it is unknown whether the reach and engagement was due to the content itself or the caption used when posting the content piece on the Facebook page. Because the post caption precedes the post content, the caption has the potential to either encourage or dissuade the Facebook user from engaging with the content.

Fourth, organic reach includes Facebook users who may not be followers of the MMHRC page, so it is possible that individuals other than women with mental health conditions who follow the MMHRC Facebook page were reached and that they engaged with the social media content.

Despite these limitations, this exploratory study is a step toward identifying and framing substance use content in a way that is accessible and available to women with mental health conditions and substance use disorders. Future studies could include additional postings to increase generalizability and perhaps repeat posting content on additional days and times to ensure all MMHRC followers can view the content.

### Conclusions and Implications

Policymakers and providers are challenged to develop effective ways to reach women with substance use disorders and engage them in services, treatment, and support that will ultimately benefit them as well as their children. Although some states offer priority treatment for this vulnerable population [35], barriers to accessing treatment persist. The COVID-19 pandemic has intensified the need for digital and, potentially, social media solutions to mitigate logistical barriers and act as an extender of the system of care to help pregnant women and mothers engage in the substance use treatment system. Although the pandemic makes it difficult for women to access in-person treatment and services due to safety concerns, online peer support, perhaps navigated via social media platforms, could reduce social isolation and result in enhanced treatment access, engagement, and outcomes for women with substance use disorders.

Social media has the potential to be an important avenue for reaching Facebook users and engaging them with substance use content, making them aware of health consequences for both the mother and baby and available treatment options. This is especially useful for engaging individuals with co-occurring mental health conditions, who can be a difficult-to-reach population for the substance use treatment system. Given the additional challenges that the COVID-19 pandemic poses in terms of the limitations of in-person treatment services and the increasing the number of individuals who may be in need of treatment for substance use disorders, these findings suggest that providers and policymakers incorporate social media in their plans to mitigate these barriers. Additional research is needed to determine why certain substances garnered more engagement than others; nevertheless, this study suggests that social media has the potential to be an equal player in the treatment system.

## Abbreviations

MMHRC: Maternal Mental Health Research Collaborative
NIAAA: National Institute on Alcohol Abuse and Alcoholism
PCORI: Patient-Centered Outcomes Research Institute
